# Five-year outcomes of posterior affected-vertebrae fixation in lumbar tuberculosis patients

**DOI:** 10.1186/s13018-018-0902-2

**Published:** 2018-08-22

**Authors:** Qiang Liang, Qian Wang, Guangwei Sun, Wenxin Ma, Jiandang Shi, Weidong Jin, Shiyuan Shi, Zili Wang

**Affiliations:** 1 Department of Spinal Surgery, General Hospital of Ningxia Medical University, 804 Shengli Street, Yinchuan, 750004 China; 2 Hillsborough Community College, Tampa, USA; 3 Department of Orthopedics, Hospital of Integrated Traditional Chinese and Western Medicine in Zhejiang Province, Hangzhou, 310003 Zhejiang China

**Keywords:** Lumbar spinal tuberculosis, Affected-vertebrae fixation, Combined posterior and anterior approach

## Abstract

**Background:**

Posterior instrumentation after deformity correction is an important method for reconstruction of spinal stability in the management of lumbar tuberculosis (TB). However, the commonly used methods include both long- and short-segment fixation of normal motor units. There has been no report regarding affected-vertebrae fixation of lumbar TB.

**Methods:**

Data from 135 patients with lumbar TB who underwent posterior instrumentation and either affected-vertebrae fixation or short-segment fixation using a combined posterior and anterior approach were retrospectively reviewed. Among these patients, 71 cases were treated with affected-vertebrae fixation, and 64 cases were treated with short-segment fixation. Debridement, bone grafting, deformity correction, and decompression were performed within all affected segments. Operative times, intra-operative blood loss, TB cure rates, bone graft fusion rates, degree of deformity correction, neurological function, pain recovery, and complications were analyzed.

**Results:**

Comparing affected-vertebrae fixation vs. short-segment fixation groups, respectively, the number of the affected segments was 107 vs. 98; average number of affected segments was 1.51 vs. 1.53; total number of fixed segments was 107 vs. 226; average number of fixed segments was 1.51 vs. 3.53; average blood loss was 726.2 ml vs. 948.5 ml; average operative time was 210.4 min vs. 270.3 min; and average hospitalization costs were 29,000 RMB vs. 42,000 RMB (all *p* values < 0.05). In the affected-vertebrae fixation vs. short-segment fixation groups, respectively, TB cure rates were 82.61% vs. 84.62% at 6 months after operation and 97.83% vs. 97.44% at 5 years after operation; bone fusion rates were 86.96% vs. 87.18% at 6 months after operation and 97.83% vs. 97.66% at 5 years after operation; average number of degrees of Cobb’s angle correction were 13.1° vs. 13.7°; average correction losses were 1.9° vs. 1.4°; and complication rates were 12.04% vs. 12.97% (all *p* values > 0.05).

**Conclusion:**

Under strict surgical indications, posterior instrumentation on affected-vertebrae is a safe, effective, and feasible fixation method in the treatment of lumber TB.

## Background

Lumbar tuberculosis (TB) accounts for approximately 50% of spinal TB. Since spinal TB can lead to deformity and neurological dysfunction in serious cases, surgery is necessary for these patients [1, 2]. Internal fixation using instrumentation is an important method of reconstructing the stability of the spine in the surgical management of spinal TB [3, 4]. Common internal fixation methods include long-segment fixation (involving two or more normal motor units superior to the affected vertebrae and two or more normal units inferior to it) and short-segment fixation (involving one normal motor unit superior to the affected vertebrae and one motor unit inferior to it) [5–7]. Although both long-segment and short-segment can restore the normal structure of the spine and maintain the correction effectively, the relatively long stretch of the fixed segments not only causes the normal motor units to lose motor function resulting in stiffness of the related segments, but also accelerates degeneration of adjacent segments [8].

In order to solve this problem, instrumentation of the affected vertebrae was proposed, which means that complete debridement [9] was performed within the affected motor units; the screws were placed into the pedicle and centrum of the affected vertebrae, the strut bone graft placed in the interval between the affected vertebrae, and decompression and deformity correction were being carried out within the interval between the affected vertebrae. The above-mentioned surgical procedure (termed affected-vertebra fixation) is performed within the affected motor units while the normal motor units are not involved, which maximally preserves motor function of the spine. However, there has been no report concerning the treatment of lumbar TB using this procedure.

A retrospective study comparing affected-vertebra fixation vs. short-segment fixation in the treatment of lumbar TB was performed with the aim to explore the feasibility of affected-vertebrae fixation and to summarize its technical points.

## Methods

### Patient data

The study protocol was approved by the Ethics Committee of the General Hospital of Ningxia Medical University and written informed consent was obtained from every subject. The present study included 135 patients with lumbar TB who underwent affected-vertebra fixation or short-segment fixation in our department between March 2007 and March 2013. Patients were selected if the three dimensional CT showed the upper and lower end plates of the affected vertebrae are intact, so as could provide a reliable host bed for the strut bone graft. Patients were excluded if they had severe kyphosis deformity (> 60°) and could not be corrected by changing the patient’s position using manual techniques and instrument application, the pedicle of vertebra had been invaded by TB, or the patients had severe osteoporosis.

Surgical indications were as follows: (1) patients with neurological dysfunction caused by spinal cord or cauda equina compression; (2) patients with spinal instability and kyphosis; (3) patients with a relatively large abscess, sequestrum, or prolonged healing of a sinus tract; and (4) patients cannot tolerate the pain caused by the lesions of lumbar tuberculosis. Patients were diagnosed with instability preoperatively when the patients meet one or more of these criterions: (1) the vertebral body was invaded by tuberculosis and the height of vertebral destruction was more than one third of the original vertebral body height; (2) vertebral spatial displacement > 3 mm; (3) lumbar kyphosis angle > 10°; (4) rotational displacement: L1,2; L2,3; L3,4 > 15°; L4,5 > 20°; L5, S1 > 25°.

All patients underwent complete debridement, bone grafting and fusion, decompression, and deformity correction within the affected segments. According to the various fixation methods, patients were divided into two groups: (1) the affected-vertebrae fixation group (*n* = 71), in which internal fixation was carried out within the affected motor units; and (2) the short-segment fixation group (*n* = 64), in which the fixation included the affected vertebrae together with one normal motor unit superior to the affected vertebrae and one inferior to it. Patient demographic and baseline variables are shown in Table 1.

**Table 1:** Preoperative patient characteristics in the two groups

Items	Affected-vertebrae fixation group	Short-segment fixation group	*p* values
Patients	71	64	
Gender m/f	32/39	31/33	*p* = 0.695
Age ($\overline{x}\pm s$, years)	41.7 ± 21.7	43.5 ± 19.6	*p* = 0.738
Disease duration ($\overline{x}\pm s$, months)	8.3 ± 4.1	7.6 ± 4.7	*p* = 0.257
Clinical manifestations			
Back pain	63	58	*p* = 0.719
Low-grade fever	52	46	*p* = 0.859
Night sweating	61	53	*p* = 0.619
Weakness	55	48	*p* = 0.737
Formation of cold abscess	32	29	*p* = 0.977
Lesion location			
L1–2	6	8	*p* = 0.880
L2–3	11	13	
L3–4	13	10	
L4–5	18	16	
L1–3	9	4	
L2–4	2	3	
L3–5	6	4	
L1–4	1	2	
L2–5	3	1	
L1–2 + L4–5	2	3	
Number of the affected segments			
Single segment	54	47	*p =* 0.903
Double segments	13	14	
Triple segments	4	3	
Cobb’s angle ($\overline{x}\pm s$, °)	24.8 ± 15.2	26.2 ± 14.1	*p* = 0.735
CRP ($\overline{x}\pm s$, mg/l)	27.0 ± 24.9	25.8 ± 22.3	*p* = 0.718
ESR ($\overline{x}\pm s$, mm/h)	36.5 ± 22.5	40.4 ± 20.1	*p* = 0.349

In this article, the application situations of the two internal fixation methods were very similar. According to the inclusive and exclusive criteria, all cases included in this study (either of the affected-vertebrae fixation group or of the short-segment fixation group) can be treated with affected-vertebrae fixation. For patients who must undergo short-segment fixation, they were excluded according to the inclusion and exclusion criteria. Because the theory and technology of affected-vertebrae fixation were not quite developed before 2010 (during the early stage of the study), we performed short-segment fixation on some patients, even though they could have met the indications of affected-vertebrae fixation. In this article, we included these cases in short-segment fixation group as a control group to evaluate the efficacy of affected-vertebrae fixation. After we have confirmed that the affected-vertebrae fixation was a safe, effective, and feasible surgical procedure through our earlier research, the affected-vertebrae fixation was implemented more often in the latter period of the study (after 2010).

### Preoperative preparation

After admission, patients were placed on strict bed rest and supportive treatment was performed. For preoperative anti-tuberculosis treatment, isoniazid (INH, H, 5 mg/kg/d), rifampicin (RFP, R, 10 mg/kg/d), pyrazinamide (PZA, Z, 20 mg/kg/d), and streptomycin (SM, S, 20 mg/kg/d) were administered for 2–4 weeks (average, 2.3 weeks). Liver function and kidney function were monitored, including liver and kidney damage being managed promptly. After the patient’s general condition had improved, surgery was carried out. A feasible surgical plan was developed for each patient according to the imaging data from CT, MRI, and ultrasonography.

### Surgical procedures

All patients underwent combined posterior-anterior surgery. Posterior pedicle screw fixation, deformity correction, and posterolateral bone grafting were carried out first and then complete anterior debridement, decompression, and strut bone grafting were performed.

### Surgical procedure in the affected-vertebrae fixation group

**Fig. 1:**
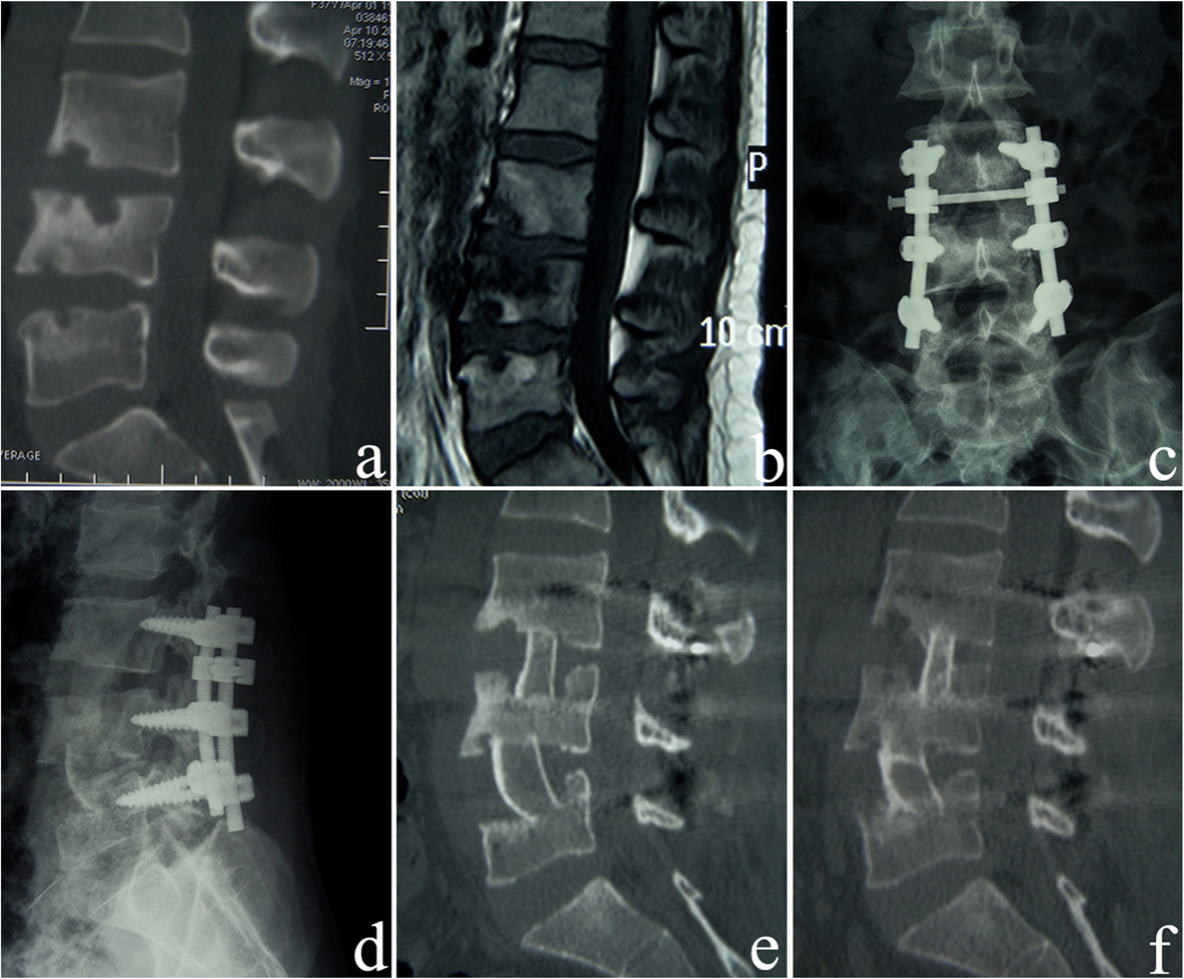
A 34-year-old female patient underwent anterior-posterior surgery and affected-vertebrae fixation. **a** The preoperative sagittal CT reconstruction image shows destruction of L3, L4, and L5 vertebrae and narrowing of the L3-L4 intervertebral space. **b** The preoperative sagittal contrast-enhanced MRI shows destruction of L3, L4, and L5 vertebrae and destruction of the L3-L4 and L4-L5 intervertebral disks. **c**, **d** One month after surgery, the anteroposterior and lateral X-ray images show that the fixation of the affected vertebrae is excellent. L3 and L4 vertebras are fixed with short pedicle screws. **e** Three months after surgery, the sagittal CT reconstruction shows that the lesion in the L3, L4, and L5 vertebrae has debrided completely, and the iliac bone grafts are firm. **f** Five years after surgery, the sagittal CT reconstruction shows that the L3–5 tuberculosis lesions are cured, and the bone graft fusion is solid

In affected-vertebrae fixation, after general anesthesia, the posterior midline approach was applied. The posterior structures of the affected segments (of the spine) were exposed by dissecting the erector spinae. Pedicle screws were placed into the pedicle of the affected vertebrae, and deformity correction was carried out using the patient’s position, manual technique, and instrument application. The principle of affected-vertebrae fixation was as follows: routine pedicle screws were placed in the affected vertebrae if the residual heights of the affected vertebrae were more than one third after debridement; short pedicle screws 25–35 mm in length were inserted if the destruction of the affected vertebrae was severe; and residual heights of the affected vertebrae were less than one third after debridement, because routine pedicle screws could not be placed. A cross bar was used for all patients to increase stability. After internal fixation, posterolateral autogenous bone grafting and fusion was applied. The posterior incision was closed after surgery via the posterior approach. Thereafter, an anterior kidney incision or a “V” incision was made use to expose the affected vertebrae via the retroperitoneal approach, and complete debridement, autologous iliac bone grafting, and anterolateral decompression were carried out. The anterior and posterior surgeries were performed at the same time or separately according to the patient’s condition (Fig. 1). In the present study, 59 out of 71 patients underwent one-stage surgery, and 12 patients underwent two-stage surgery.

### Surgical procedure in the short-segment fixation group

**Fig. 2:**
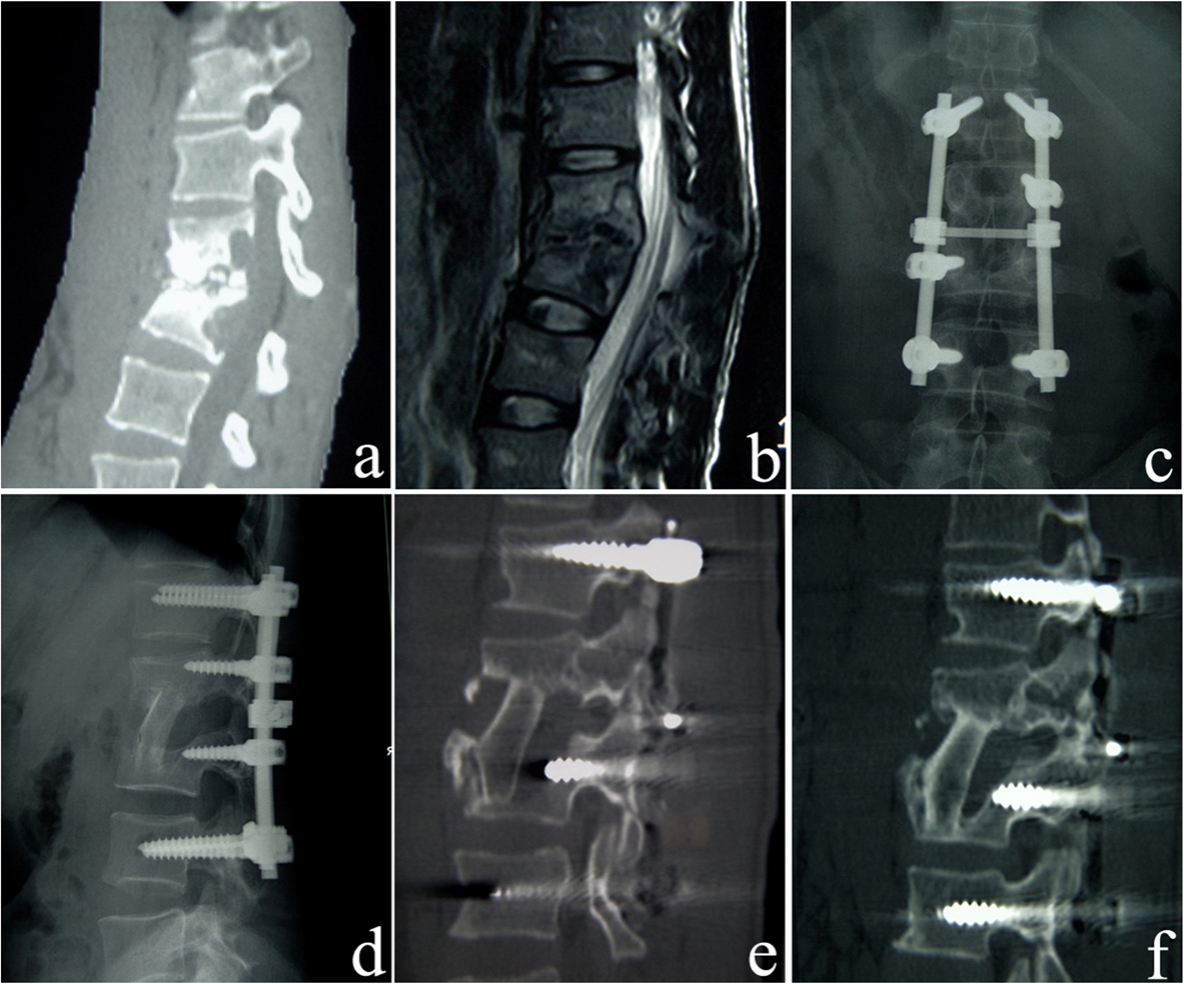
A 25-year-old male who underwent anterior-posterior surgery and short-segment fixation. **a** Preoperative sagittal CT reconstruction shows destruction of L2 and L3 vertebrae and narrowing of the L2-L3 intervertebral space. **b**. The preoperative sagittal contrast-enhanced MRI shows confounding signals in L2 and L3 vertebrae, destruction of the L2-L3 intervertebral space, and nerve compression. **c**, **d** Postoperative X-ray anteroposterior image shows that the strut bone is located firmly between the affected vertebrae, the short-segment fixation is excellent, and the L2 and L3 vertebrae are fixed with short pedicle screws. **e** Three months after surgery, the sagittal CT reconstruction shows that the lesion of L2 and L3 vertebrae is removed completely, and the strut bone is located firmly. **f** Five years after surgery, the sagittal CT reconstruction shows healing of L2 and L3 tuberculosis lesions and bone graft fusion

The surgical approach and basic steps of short-segment fixation were the same as those of the affected-vertebrae fixation. The only difference between the two procedures involved is the placement of the pedicle screws, which were not only placed in the affected vertebrae, but also inserted in the normal motor units (superior and inferior to the affected vertebrae, one for each side) in short-segment fixation. A cross bar was also used for all patients to increase stability. However, posterolateral bone grafting and anterior strut bone grafting were the same as those of affected-vertebrae fixation (Fig. 2). In the short-segment fixation group, 45 out of 64 patients underwent one-stage surgery, and 19 patients underwent two-stage surgery.

### Postoperative treatment and follow-up

A drainage tube was placed in the surgical wound under negative pressure, and the tube was removed when the volume of drainage was < 20 ml per day. The patient was placed on bed rest for 2–3 weeks after surgery and then ambulating with orthosis was started. Waist flexion, overextension, lateral bending, and rotation were avoided before bone graft fusion. A 2HRZS/2-xHRZ regimen was used for chemotherapy; the duration of intensive chemotherapy was 2 months and that of consolidation chemotherapy was 2–7 months [10, 11]. Both the patient’s follow-up and chemotherapy management were performed by a specifically assigned doctor. For the 6 months after surgery, each patient visited our hospital once every month, and from 6 months to 5 years after surgery, the patient visited the hospital once a year. Follow-up included medical history and physical exam, erythrocyte sedimentation rate (ESR), C-reactive protein level, liver function panel, renal function panel, X-rays, CT reconstruction, MRI, and ultrasonography. When these indicators normalized, the healing of the lesion was determined based on the imaging findings, and a decision regarding drug withdrawal was made.

We used the Cobb’s angle to evaluate the sagittal deformity of spinal tuberculosis. The Cobb’s angle was measured by drawing lines along the uppermost and lowermost endplate of the affected segment in lateral radiograph. An initial halo sign (radiolucent line around the implant > 1 mm wide) followed by a double halo sign on later radiographs or CT scans was defined as screw loosening. Bone graft fusion was determined based on a three-dimensional CT reconstruction using the following parameters: no displacement or tilting of the bone graft, no absorption or hardening of the boundary between the bone graft and the host bed, the bone graft was in close contact with the graft bed, and there was trabecular bone formation, bone bridge connection, and reshaping in the blurred boundary. Standards used to determine the healing of spinal TB included an excellent general condition of the patient, lack of local pain or tenderness, no cold abscess or sinus formation, ESR and CRP levels which were continuously normal, and imaging which the data showed complete bone graft fusion without new TB lesions.

### Statistical analysis

Statistical analysis was performed using the SPSS 22.0 (SPSS, USA) statistical software package. Differences in age, follow-up time, VAS (visual analogue scale) score, volume of blood loss, operative time, hospitalization cost, CRP, ESR, and Cobb’s angle between the two groups were compared using the Student’s *t* test (data conformed to normal distribution by Shapiro-Wilk test) or non-parametric test (data not conformed to normal distribution by Shapiro-Wilk test). Gender, clinical symptoms, the number of affected segments, the number of fixed segments, bone graft fusion rate, the rate of TB healing, and the incidence of complications were compared by χ^2^ test. A statistical significance level of 0.05 was adopted.

## Results

All patients were followed up for more than 5 years. The average intraoperative blood loss of the affected-vertebrae fixation group vs. short-segment fixation group, respectively, was 726.2 ml vs. 948.5 ml (*t* = − 18.57, *p* = 0.000), average operative time was 210.4 min vs. 270.3 min (*t* = − 7.94, *p* = 0.000), and average hospitalization cost was 29,000 RMB vs. 42,000 RMB (*t* = − 2.65, *p* = 0.009) (Table 2).

**Table 2:** Operative time, intraoperative blood loss, and hospitalization cost in the affected-vertebrae fixation group and short-segment fixation group ($\overline{x}\pm s$)

Groups	Number of patients	Operation time (min)	Intraoperative blood loss (ml)	Hospitalization cost (10,000 RMB)
Affected-vertebrae fixation group	71	210.4 ± 36.8	726.2 ± 60.3	2.9 ± 2.6
95% CI		201.8 to 219.0	712.2 to 740.2	2.3 to 3.5
Short-segment fixation group	64	270.3 ± 50.4	948.5 ± 78.4	4.2 ± 3.1
95% CI		257.9 to 282.7	929.3 to 967.7	3.4 to 5.0
*p* value		0.000	0.000	0.006

In the affected-vertebrae fixation group vs. short-segment fixation group, respectively, the total number of affected segments was 107 vs. 98, average number of affected segments was 1.51 vs. 1.53, total number of fixed segments was 107 vs. 226, and average number of fixed segments was 1.51 vs. 3.53 in each case. The fixed segments of the affected-vertebrae fixation group were 2.02 segments less than those of the short-segment fixation group in each case. In the affected-vertebrae fixation group vs. short-segment fixation group, respectively, the average number of degrees of Cobb’s angle correction was 13.1° vs. 13.3°, while the mean correction loss was 1.9° vs. 1.4°, (*p* > 0.05) (Table 3). At the last follow-up, the TB healing rates and bone graft fusion rates were > 98% in the two groups (Table 4), and the differences were not significant. Six months after surgery, ESR and CRP were reduced to normal levels in both groups (Table 5).

**Table 3:** Postoperative recovery of Cobb’s angle in the affected-vertebrae fixation group and short-segment fixation group ($\overline{x}\pm s$)

Groups	No. of patients	Before operation (°)	After operation (°)	Last follow-up (°)	Correction (°)	Loss (°)
Affected-vertebrae fixation group	71	24.8 ± 15.2	8.9 ± 3.5	10.8 ± 3.8	15.9 ± 10.2	1.9 ± 0.6
95% CI		21.3 to 28.3	8.1 to 9.7	9.9 to 11.7	13.5 to 18.3	1.8 to 2.0
Short-segment fixation group	64	26.2 ± 14.1	9.5 ± 4.1	11.1 ± 4.3	17.7 ± 9.5	1.6 ± 1.7
95% CI		22.7 to 29.7	8.5 to 10.5	10.0 to 12.2	15.4 to 20.0	1.2 to 2.0
*p* value		0.735	0.361	0.668	0.326	0.174

**Table 4:** Disease healing and bone graft fusion in the affected-vertebrae fixation group and short-segment fixation group

Groups	Cases	Lesion cured (cases)			Bone graft fusion (cases)		
		6 months after surgery	1 year after surgery	5 years after surgery	6 months after surgery	1 year after surgery	5 years after surgery
Affected-vertebrae fixation group	71	57	69	71	54	68	71
Short-segment fixation group	64	52	61	64	44	57	64
*p* value		0.887	0.566	–	0.342	0.137	–

**Table 5:** Preoperative and postoperative ESR and CRP in the affected-vertebrae fixation group and short-segment fixation group ($\overline{x}\pm s$)

Groups	Cases	Before surgery		6 months after surgery		5 years after surgery	
		ESR (mm/h)	CRP (mg/l)	ESR (mm/h)	CRP (mg/l)	ESR (mm/h)	CRP (mg/l)
Affected-vertebrae fixation group	71	36.5 ± 22.5	27.0 ± 24.9	16.6 ± 4.2	2.2 ± 1.7	9.3 ± 2.4	1.4 ± 1.1
95% CI		31.3 to 41.7	21.3 to 32.7	15.6 to 17.6	1.8 to 2.6	8.7 to 9.9	1.1 to 1.7
Short-segment fixation group	64	40.4 ± 20.1	25.8 ± 22.3	17.4 ± 3.7	2.4 ± 2.3	8.6 ± 3.5	1.1 ± 1.5
95% CI		35.5 to 45.3	20.3 to 31.3	16.5 to 18.3	1.8 to 3.0	7.7 to 9.5	0.7 to 1.5
*p* value		0.349	0.718	0.245	0.498	0.174	0.164

The normal range for ESR: male 0–15 mm/h, female 0–20 mm/h; the normal range for CRP 0–2.87 mg/l

*CRP* C-reactive protein, *ESR* erythrocyte sedimentation rate

In the affected-vertebrae fixation group vs. short-segment fixation group, respectively, average preoperative VAS score (visual analogue scale pain score) was 7 (range, 6–8) vs. 6.5 (range, 5–8), while the average score at the last follow-up was 1 (range, 0–2) vs. 1.5 (range, 1–3). The neural function of both groups improved significantly after surgery, and the outcomes were satisfactory (Table 6). In the affected-vertebrae fixation group vs. short-segment fixation group, respectively, the preoperative JOA (JOA = Japanese Orthopedic Association) score was 14.43 vs. 14.25 (*t* = 0.520, *p* = 0.405), the postoperative JOA score was 27.93 and 27.74 (*t* = 1.086, *p* = 0.415), and the improvement rate was 92.66% vs. 91.46% (*χ*^*2*^ = 0.098, *p* = 0.754).

**Table 6:** Preoperative and postoperative ASIA grade in the affected-vertebrae fixation group and short-segment fixation group

Grades	Affected-vertebrae fixation group (*n* = 71)						Short-segment fixation group (*n* = 64)					
	Before surgery	After surgery					Before surgery	After surgery				
		A	B	C	D	E		A	B	C	D	E
A	0						0					
B	2			1	1		3			1	1	
C	5				2	3	6				2	4
D	36				2	34	34				1	33
E	28					28	21					21

*ASIA grade* American Spinal Injury Association grade

No damage occurred to the spinal cord, cauda equina, nerve roots, large blood vessels, or important organs in any of these patients. In four cases, fat liquefaction occurred at the anterior incisions, which healed after 3 weeks of dressing. In two cases (one in each group) debridement was incomplete, and TB was not cured. After 2 months, a psoas muscle abscess appeared, and secondary surgery was performed. Both patients were treated successfully. In two cases (one in each group), the loosening of internal fixation occurred at 3 months and 5 months after surgery, and they quickly underwent revision surgery. In three cases (two cases in the affected-vertebrae fixation group and one case in the short-segment fixation group), the strut bone graft was tilted, and both time of immobilization and duration of anti-TB treatment were prolonged. Bone graft fusion was achieved thereafter.

## Discussion

Lumbar TB has the highest incidence of spinal TB, and surgical intervention combined with anti-TB chemotherapy can ensure good outcomes and treat paralysis [12–14]. Internal fixation is a necessary method for reconstruction of spinal stability. At present, short- and long-segment fixations are commonly used for internal fixation during surgical treatment of spinal TB [15, 16]. These methods not only provide strong fixation, but also have a certain impact on the structure and motor function of the spine.

The range of movement in each normal motor unit of the lumbar spine includes 10–15°of flexion and extension, 6–8°of lateral bending, and 2°of axial rotation, while the short-segment and long-segment fixations restrict at least two normal motor units. In addition, if the range of fixation is too long, degenerative changes may easily occur in adjacent segments. Biomechanical studies and clinical observations suggest that the longer the fixed fusion segment, the greater the activity of the adjacent segments and the greater the pressure on the intervertebral disc, the more likely the chances that degeneration will occur in the adjacent segments [17, 18]. Affected-vertebrae fixation can carry out debridement, decompression, deformity correction, bone grafting, and internal fixation within the affected motor units without involving adjacent normal motor units, thus, maximizing the retention of spinal motion function and reducing adjacent segmental degeneration.

The results of this study show that compared with the short-segment fixation group, the affected-vertebrae group had shorter operative times, less intraoperative blood loss, and because fixation was limited to the affected motor units, as well as the average number of fixation segments being reduced by 2.02. However, there was no significant difference between the two groups with regards to the VAS score, ASIA impairment scale, ESR, CRP, lesion cure rate, bone graft healing rate, or incidence of complications. In addition, there was no significant difference between the two groups with regards to deformity correction, average postoperative correction in Cobb’s angle, and loss of correction at 5 years after surgery. The degree of correction in Cobb’s angle and loss of correction in the affected-vertebrae group were similar to the results of Wang et al. [19] and Mukhtar et al. [20], who studied long-segment vs. short-segment fixation and reported a loss of correction of < 3°. Therefore, affected-vertebrae fixation, used in the treatment of lumbar TB, can safely and effectively reconstruct spinal stability and maintain the correction of deformity for long periods.

During an earlier study, we produced a reconstruction model with iliac strutting in the anterior and mid column, affected-vertebrae fixation in calf spine. The ranges of motion (ROM) of intact spines were tested as control. The ROM in axial compression, 1ateral bending, flexion-extension, and rotation were tested. No statistical difference of the ROM was found between affected-vertebrae fixation group and control group (*p* > 0.05). So we concluded that with iliac strutting in the anterior and mid column, affected-vertebrae fixation can provide instant stability [21]. The above-mentioned biomechanical study provides a strong theoretical basis for clinical implementation of affected-vertebrae fixation. In clinical studies, Xu et al. [22] and Liu et al. [23] treated thoracolumbar fractures with single-segment fixation and obtained satisfactory outcomes without complications such as internal fixation breakage or loosening. These studies provide the biomechanical and clinical bases for affected-vertebrae fixation in the treatment of spinal TB.

Although the mechanical properties of affected-vertebrae fixation are less rigid than those of short-segment fixation and long-segment fixation, we found that an appropriate technique can meet the requirement of rigid fixation. Therefore, it is very important to define strict indications for surgery. We summarized the indications and contraindications of affected-vertebrae fixation as follows. The indications of affected-vertebrae fixation are as follows: (1) the upper and lower end plates of the affected vertebrae were intact showed by preoperative computer tomography (CT) scan and three-dimensional (3D) CT imaging reconstruction, so as to provide a reliable host bed for the strut bone graft; (2) the kyphosis deformity was not serious (< 60°) and could be corrected by changing the patient’s position using manual techniques and instrument application; (3) the pedicle should be relatively intact without TB invasion. The contraindications of affected-vertebrae fixation are as follows: (1) patients with severe osteoporosis; and (2) bony-diseases cured or bony-stationary of spinal tuberculosis need to be treated with corrective osteotomy. The affected-vertebrae fixation will be implemented once the patient meets the criteria for affected-vertebrae fixation, as it is less invasive and the cost is lower. For those patients with (1) the pedicle of the affected vertebrae was invaded by TB, (2) the kyphosis deformity was serious (> 60°), (3) severe osteoporosis, and (4) bony-diseases cured or bony-stationary of spinal tuberculosis, we will consider to give them a short-segment fixation in our clinic work now.

Affected-vertebrae fixation is one component of affected-vertebrae surgery, which requires removal of the lesion, decompression, deformity correction, bone grafting, and fixation within the affected vertebrae. The application of posterior affected-vertebrae fixation can stabilize the posterior column, but there is a relatively large bone defect in the anterior and middle columns after anterior debridement, which results in instability in the anterior and middle columns. Therefore, strut bone grafting should be carried out to support the anterior and middle columns for successful fixation of the affected vertebrae. Strut bone grafting can reduce the load of posterior fixation devices in the corresponding segments, reduce stress, and protect internal fixation devices [24]. After debridement, the host bed for bone grafting should be trimmed evenly to produce an adequate condition for the survival of the bone graft. The strut bone should be placed perpendicular to the upper and lower end plates and placed as close to the center as possible to avoid loss of correction and difficulty in bone fusion due to uneven loads. Moreover, our previous biomechanical study showed that the crosslink could increase the anti-torsion ability of the screw-rod system, reduce stress on the posterior articular process, and enhance axial stability of the spine. Therefore, it should not be disregarded in the process of posterior affected-vertebrae fixation.

## Conclusion

In conclusion, based on chemotherapy requirements and using strict surgical indications, posterior affected-vertebrae fixation (together with anterior debridement and bone grafting) is a safe, effective, and feasible surgical procedure in the treatment of lumbar TB. In addition, it is less invasive, and the cost is lower. However, the present study is a single-center study, and the sample size is relatively small. Therefore, multicenter, large-sample, prospective randomized controlled studies should be carried out in the future to improve the level of evidence-based medicine and support our findings.

## Abbreviations

ASIA: American Spinal Injury Association
CRP: C-reactive protein
CT: Computed tomography
ESR: Erythrocyte sedimentation rate
HRZS: Isoniazid (H), rifampicin (R), pyrazinamide (Z), chain toxin (S)
MRI: Magnetic resonance imaging
TB: Tuberculosis
VAS: Visual analogue scale

## References

[1] Gao Y, Ou Y, Deng Q, He B, Du X, Li J (2017). Comparison between titanium mesh and autogenous iliac bone graft to restore vertebral height through posterior approach for the treatment of thoracic and lumbar spinal tuberculosis. PLoS One.

[2] Kandwal P, Jayaswal A, G V (2016). Management of tuberculous infection of the spine. Asian Spine J.

[3] Hassan K, Elmorshidy E (2016). Anterior versus posterior approach in surgical treatment of tuberculous spondylodiscitis of thoracic and lumbar spine. Eur Spine J.

[4] Huang Y, Lin J, Chen X, Lin J, Lin Y, Zhang H (2017). A posterior versus anterior debridement in combination with bone graft and internal fixation for lumbar and thoracic tuberculosis. J Orthop Surg Res.

[5] Pang X, Wu P, Shen X, Li D, Luo C, Wang X (2013). One-stage posterior transforaminal lumbar debridement, 360° interbody fusion, and posterior instrumentation in treating lumbosacral spinal tuberculosis. Arch Orthop Trauma Surg.

[6] Wang Y, Zhang H, Tang M, Guo C, Deng A, Wu J (2016). One-stage posterior focus debridement, interbody grafts, and posterior instrumentation and fusion in the surgical treatment of thoracolumbar spinal tuberculosis with kyphosis in children: a preliminary report. Childs Nerv Syst.

[7] Zeng H, Wang X, Zhang P, Peng W, Liu Z, Zhang Y (2015). Single-stage posterior transforaminal lumbar interbody fusion, debridement, limited decompression, 3-column reconstruction, and posterior instrumentation in surgical treatment for single-segment lumbar spinal tuberculosis. Acta Orthop Traumatol Turc.

[8] Wang H, Ma L, Yang D, Wang T, Liu S, Yang S (2017). Incidence and risk factors of adjacent segment disease following posterior decompression and instrumented fusion for degenerative lumbar disorders. Medicine (Baltimore).

[9] Shi J, Wang Q, Wang Z (2014). Primary issues in the selection of surgical procedures for thoracic and lumbar spinal tuberculosis. Orthop Surg.

[10] Wang Z, Ge Z, Jin W, Qiao Y, Ding H, Zhao H (2007). Treatment of spinal tuberculosis with ultrashort-course chemotherapy in conjunction with partial excision of pathologic vertebrae. Spine J.

[11] Wang Z, Shi J, Geng G, Qiu H (2013). Ultra-short-course chemotherapy for spinal tuberculosis: five years of observation. Eur Spine J.

[12] Rajasekaran S, Kanna RM, Shetty AP (2015). History of spine surgery for tuberculous spondylodiscitis. Unfallchirurg.

[13] Jin W, Wang Q, Wang Z, Geng G (2014). Complete debridement for treatment of thoracolumbar spinal tuberculosis: a clinical curative effect observation. Spine J.

[14] Jiang T, Zhao J, He M, Wang K, Fowdur M, Wu Y (2015). Outcomes and treatment of lumbosacral spinal tuberculosis: a retrospective study of 53 patients. PLoS One.

[15] Alam MS, Phan K, Karim R, Jonayed SA, Munir HK, Chakraborty S (2015). Surgery for spinal tuberculosis: a multi-center experience of 582 cases. J Spine Surg.

[16] Gong K, Wang Z, Luo Z (2011). Single-stage posterior debridement and transforaminal lumbar interbody fusion with autogenous bone grafting and posterior instrumentation in the surgical management of lumbar tuberculosis. Arch Orthop Trauma Surg.

[17] Park P, Garton HJ, Gala VC, Hoff JT, McGillicuddy JE (2004). Adjacent segment disease after lumbar or lumbosacral fusion: review of the literature. Spine (Phila Pa 1976).

[18] Virk SS, Niedermeier S, Yu E, Khan SN (2014). Adjacent segment disease. Orthopedics.

[19] Wang X, Pang X, Wu P, Luo C, Shen X (2014). One-stage anterior debridement, bone grafting and posterior instrumentation vs. single posterior debridement, bone grafting, and instrumentation for the treatment of thoracic and lumbar spinal tuberculosis. Eur Spine J.

[20] Mukhtar AM, Farghaly MM, Ahmed SH (2003). Surgical treatment of thoracic and lumbar tuberculosis by anterior interbody fusion and posterior instrumentation. Med Princ Pract.

[21] Wu QJ, Wang ZL, Ge CH (2010). Biomechanical test of interbody bone graft in spinal tuberculosis constructed by monosegment fixation. NingXia Med J.

[22] Xu G, Fu X, Du C, Ma J, Li Z, Tian P (2014). Biomechanical comparison of mono-segment transpedicular fixation with short-segment fixation for treatment of thoracolumbar fractures: a finite element analysis. Proc Inst Mech Eng H.

[23] Liu L, Gan Y, Zhou Q, Wang H, Dai F, Luo F (2015). Improved monosegment pedicle instrumentation for treatment of thoracolumbar incomplete burst fractures. Biomed Res Int.

[24] Talu U, Gogus A, Ozturk C, Hamzaoglu A, Domanic U (2006). The role of posterior instrumentation and fusion after anterior radical debridement and fusion in the surgical treatment of spinal tuberculosis: experience of 127 cases. J Spinal Disord Tech.

